# Water source quality testing in Gezira State, Sudan, using the compartment bag test

**DOI:** 10.1007/s13201-019-1079-5

**Published:** 2019-10-22

**Authors:** Eltigani Bashier Abdelgalili, Mohamadani Ahmed, Jaafar Adam, Samira Hamid, Traore Afsatou, Ibtisam Elshiekh, Potgieter Natasha

**Affiliations:** 1 Water Management and Irrigation Institute (WMII), University of Gezira, Wad Madani, Sudan; 2 Faculty of Medicine, University of Gezira, Wad Madani, Sudan; 3 Blue Nile National Institute for Communicable Diseases (BNNICD), University of Gezira, Wad Madani, Sudan; 4 Microbiology Department, School of Mathematical and Natural Sciences, University of Venda, Thohoyandou, South Africa; 5 Academy of Health Sciences, Wad Madani, Gezira State Sudan

**Keywords:** Compartment bag test, Gezira State, Sudan, Water sources quality

## Abstract

Although poor water quality is recognized as a public health threat, it has been little investigated in Sudan. In this paper, water sources in Gezira State, Greater *Wad Medani* locality, have been categorized as safe, intermediate safe and high-risk unsafe sources using the compartment bag test (CBT) to detect *E. coli*, which is an indicator of fecal contamination of water. The CBT is simple, portable and self-contained, and it can be done in the field environment. A total of 122 samples were collected from different water sources and included rivers, water treatment plant, boreholes/tube wells, hand pumps, public water taps, public water coolers, public elevated water tanks and household elevated water tanks. It was found that 69% (84/122) of investigated water sources were safe to drink. The sources most likely to be contaminated were those close to industrial points and factories or open sources exposed to pollution. The result showed that the highest level of contamination of water sources (high risk and unsafe) was observed in rural area (9.1%) followed by urban (5.7%) and peri-urban (1.6%). Frequent and routine qualitative analysis of water sources using CBT is recommended to improve human health and hence the country’s development.

## Introduction

Drinking water in developing countries is irregularly tested and monitored for quality because it is extremely difficult and costly to monitor water quality in low-resource settings. Globally, water quality is recognized as one of the greatest threats to human health (Cairncross 2013). UNICEF and WHO reported that over 780 million people are still lacking access to safe sources of drinking water (UNICEF/WHO 2012). For several decades, about a billion people in developing countries have not had a safe water supply (Hunter et al. 2010).

Contaminated drinking water, along with inadequate supplies of water for personal hygiene and poor sanitation, are the main contributors to an estimated 4 billion cases of diarrhea each year causing 2.2 million deaths, mostly among children under the age of five (Clasen and Bastable 2003). An estimated 884 million people are drawing their drinking water from lakes and rivers, unprotected wells and springs, and other sources that are often highly contaminated with waterborne pathogens (Rosa and Clasen 2010). In sub-Saharan Africa, 42% of the population is without improved water, 64% is without improved sanitation, and deaths due to diarrheal diseases are greater than in any other region (Montgomery and Elimelech 2007). Barbieri et al. (2019) assessed the groundwater quality in the buffer zone of Limpopo National Park and found that only 13.3% of the groundwater was fresh and suitable for drinking purposes; the remaining 86.6% was brackish and undrinkable.

Unsafe water and poor sanitation and hygiene have been reported to rank third among the 20 leading risk factors for health burden in developing countries, including Sudan (Shanan et al. 2015). In Sudan where adequate quantities of domestic water are already available on demand, the main task over the next few years will be to sustain water quality. Eltigani et al. (2015) concluded that greater qualitative efforts are needed to make water sources safe to use. Although Sudan is a country particularly rich in water resources, the quality and safety of its drinking water sources have not been fully investigated and are therefore largely unknown.

It has been reported that access to water and sanitation are extremely low in rural areas in Sudan (USAID 2009). In Gezira, more than 70% of populations in the villages are infected by waterborne diseases mainly because of the use of the polluted irrigation canal water for their domestic water needs (Henri et al. 2002).

The surface water quality is a matter of serious concern today (Singh et al. 2005). Water sources used in Sudan are diverse. The Blue Nile and groundwater are the most important water sources in Sudan and Gezira State particularly. Other sources are directly or indirectly linked with rivers and groundwater. These include water taps, elevated tanks, public water pot taps, boreholes, irrigation canals and hand pumps. The risk posed by each of these sources varies greatly. Approximately 13.5% prevalence of diarrheal diseases in Sudan was recorded among children living in areas where people draw water from uncertified *Hafirs* (Shanan et al. 2015). Both sources present health hazards to the users. Sudan has six irrigation schemes. Expansion of irrigation systems has adverse impacts on quality of water resources (Valipour 2014). Murcott, et al. (2015) stated that in both humanitarian emergency and development settings, bacteriological water quality testing is essential as it provides vital information on the microbial safety of drinking water resources. Zamxaka et al. (2004) confirmed that the evaluation of potable water supplies for coliform bacteria is important in determining the sanitary quality of drinking water.

Little work has been done on microbial quality of the drinking water in Sudan. Musa et al. (1999) investigated water quality in northern Sudan and found that fecal coliform counts grossly in excess of WHO standards. Ell-Amin et al. (2010) have carried out microbiological tests on the drinking water quality of Wad Medani and found that both surface and groundwater were highly contaminated with total coliform and fecal coliform. In addition, El Karim et al. (1985) conducted a study in Sudan and found that all water sources were invariably contaminated with coliforms. Abdelrahman and Eltahir (2011) found that in South Darfur (Sudan) the highest level of contamination of water sources was observed in household storage containers (20%) followed by boreholes (11.25%), reservoirs (6.24%), hand pumps (5.42%) and dug wells (2.49%). Generally, these water sources have been used without any routine qualitative analysis.

*Escherichia coli* are present because of breaks in water lines, uncovered sources and mismanagement of sources. The present study focused on *E. coli* in water sources using the CBT. Categorization of water sources using WHO risk guidelines to safe and unsafe water will help pinpoint risky sources and hence assist in improving community health.

## Materials and methods

### Description of study area

**Fig. 1:**
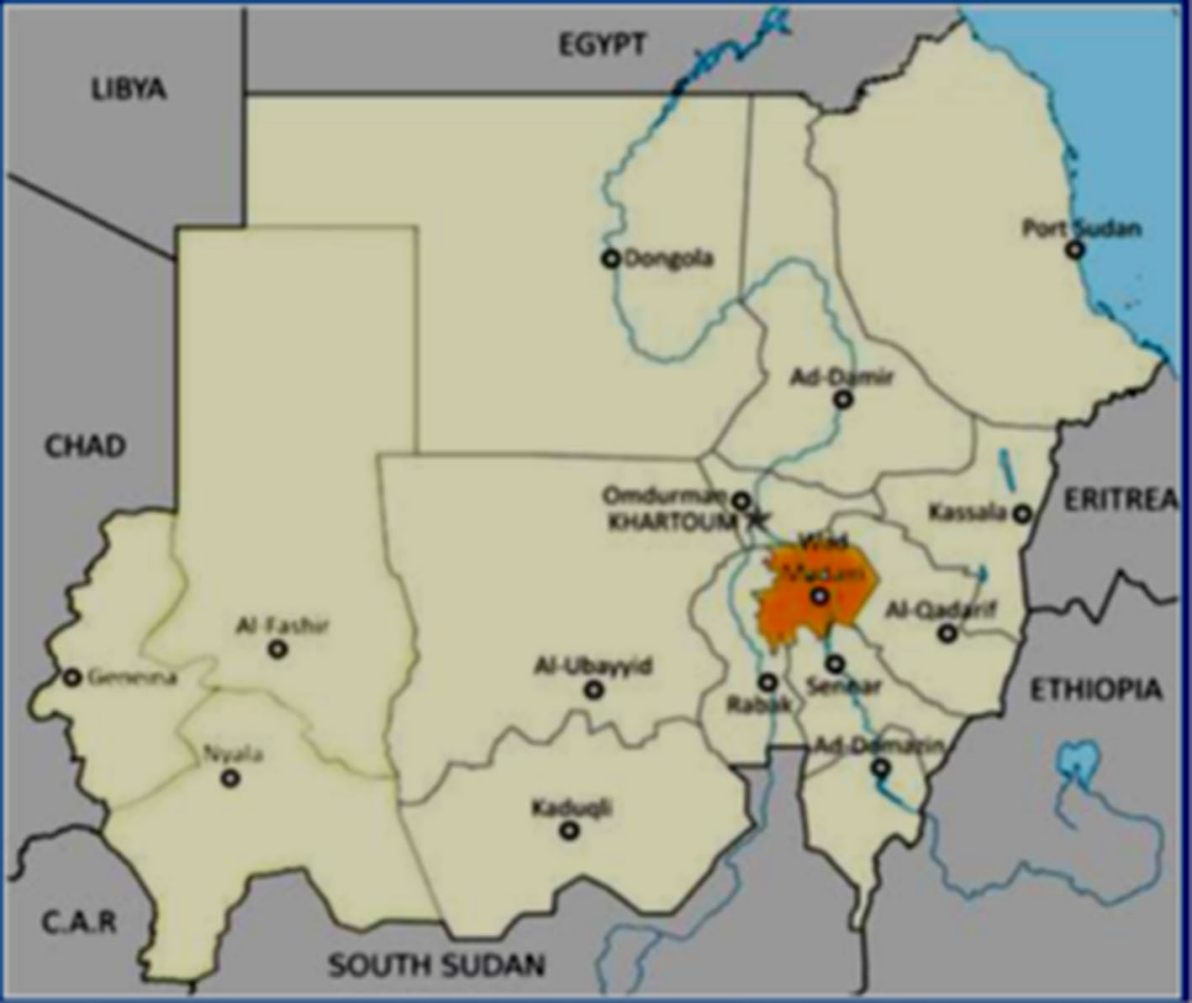
Gezira State, Sudan (Eltigani et al. 2015)

Gezira State is located in the central part of Sudan. It is bounded by latitudes 14°24′04″N and 33°31′11″E (Kogan 1990) (Fig. 1). The state is considered to be one of the most densely populated with a total population of 3.5 million people (53% rural, 35% urban and 12% nomad) with an annual growth rate of 2.9% (Sudan population census 2008, 2010; Gezira Population Statistical Office). Rainfall in Gezira ranges from 250 to 350 mm, and the soil is predominantly clay (Elias et al. 2001). Most of the rural populations are agro-pastoralists, according to the 5th Sudan population and housing census held in 2008. The state is surrounded by the White Nile and Dinder Rivers and traversed by the Blue Nile and Rahad Rivers. The state is divided into seven localities (administrative subdivisions). This study was conducted at Greater Wad Medani locality which included urban, peri-urban and rural communities. *Wad Medani* city is the capital of the state. The mean monthly temperature in the state is about 22 °C in January and 34 °C in May. Humidity varies from 13% in the north to more than 60% in the southern part of the state.

### Sample collection

Samples were collected to represent the whole study area. Samples were collected during June and July 2016 where temperature ranged from 40.3 to 25.5 °C maximum and minimum, respectively. A total number of 122 samples were collected from different sources and included river, water treatment plant, boreholes, hand pumps, public water taps, public water pots, public water coolers, public water tanks and household water tanks. Table 1 provides a basic description of all water sources assessed in this study.

**Table 1:** Water sources characteristics

Source	Samples number	Characteristic	Photograph
Tap water	53	Taps connect water from the public network to household (yard tap, communal tap, household tap)	
Public tank	11	A big tank with capacity of about 10,000–15,000 L. It is used to distribute water to a group of households	
Public cooler	9	A water cooler normally located in the market and some institutions/work places to be used by the workers, or in the market to be used by a number of people	
Hand pumps/boreholes	39	A water well normally exists in rural and peri-urban areas. It is operated manually and discharges about 5 L of water per minute.	
Treatment plant	1	Water treatment station to treat the water before distribution in the network	
Irrigation canals	3	Canals deliver water from reservoirs to the farms	
River	1	The Blue Nile River	
Bottled water/vendor water	5	Bottled water is drinking water packaged in bottle or glass water bottles. Size is small single serving bottles	

### Physical parameters of water

Temperature, pH and turbidity were measured for each sample collected. Temperature was measured using a thermometer (76-mm immersion, made in UK). Temperature will have an impact on the acceptability of a number of other inorganic constituents and chemical contaminants that may affect taste. High water temperature enhances the growth of microorganisms and may increase problems related to taste, odor, color and corrosion.

A pH meter (Sension 3 Millimeter, HACH) was used to measure pH value in all samples collected. High pH causes a bitter taste; biological contamination can change a waters pH, which in turn can harm animals and plants living in the water (Patil et al. 2012).

Turbidity was measured in nephelometric turbidity units (NTU). According to the WHO guidelines the 5th edition, turbidity must be at the level up to 5 NTU (WHO 2012). Turbidity consists of suspended material in water, causing a cloudy appearance. The suspended matter may be inorganic or organic. Turbidity in surface waters may be the result of particulate matter of many types and is more likely to include attached microorganisms that are a threat to health (WHO 2011).

### Water sample assessment using the compartment bag test

**Fig. 2:**
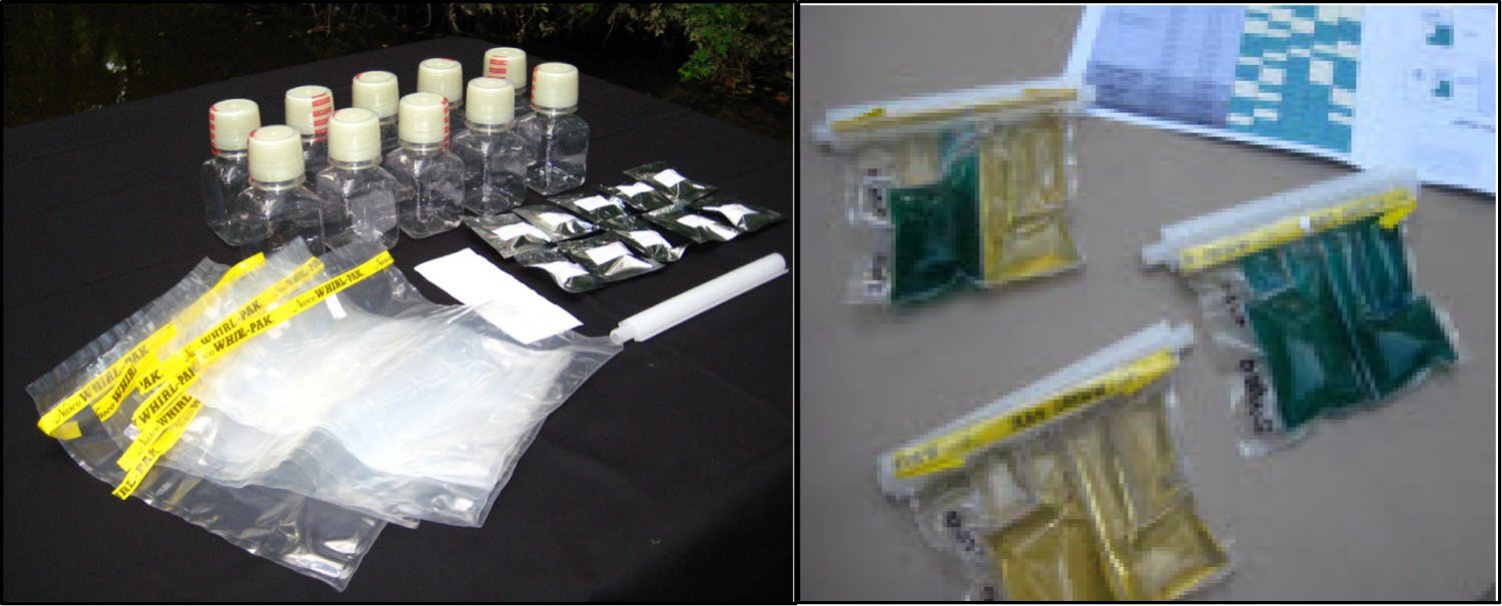
Compartment bag (Stauber et al. 2014)

CBT is simple, portable and self-contained, and it can be done in a field environment. For each water sample, the supplied *E. coli* chromogenic medium was dissolved in 100 mL of the water sample after which the sample was poured into the specially designed 100-mL compartment bag and incubated at 37 °C for 24 h in an incubator (MIR-153, SANIO). Each compartment bag is divided into 1-mL, 3-mL, 10-mL, 30-mL and 56-mL compartments (Fig. 2). Distilled sterile water was used for negative control and water containing *E. coli* as positive control. CBT calculated the most probable number (MPN) of *E. coli* per each 100 mL of water. Results were registered using WHO standards (Table 2).

**Table 2:** Health risk based on WHO guidelines for drinking water quality (WHO 2011)

Health risk category	*E. coli*/100 mL
Low risk/safe	0
Intermediate risk/probably safe	1–10
High risk/probably unsafe	11–100
Very high risk/unsafe	Greater than 100

### Statistical analysis

All water assessment data were entered into a Microsoft Excel spreadsheet. After cleaning of the data, it was exported to Stata 14 statistical package for data analysis. Percentages, frequencies and cross tables were used to discuss the results.

## Results and discussion

### Physical parameters of water sources

In this study, the parameters included pH, turbidity and temperature that may indicate changes in water quality. A pH meter was used to measure pH of collected samples in the field. The results showed a mean range from 7.4 to 7.8 for all sources (Table 3), which indicated that the water sources were neutral.

**Table 3:** Physical parameters of sources

Water source	pH				Temperature (°C)				Turbidity (NTU)			
	Mean	Min	Max	SD	Mean	Min	Max	SD	Mean	Min	Max	SD
Yard tap (*n* = 4)	7.5	7.3	7.7	0.1	25.7	25.0	26.7	0.7	11.2	0	30.6	14.0
Communal tap (*n* = 9)	7.4	7.3	7.6	0.1	25.1	24.3	25.5	0.4	1.1	0	5.6	1.8
Household tap (*n* = 40)	7.5	7.0	7.9	0.2	25.3	23.9	26.1	0.4	2.4	0	40.1	7.8
Tank water (*n* = 11)	7.4	7.1	7.8	0.2	25.2	25.0	25.7	0.2	0.5	0.1	3.0	0.9
River water (*n* = 1)	7.8	7.8	7.8	–	25.5	25.5	25.5	–	5500	5500	5500	–
Borehole water (*n* = 39)	7.4	7.0	7.8	0.2	25.3	24.9	25.8	0.3	0.8	0	28	4.5
Vendor water (*n* = 5)	7.4	7.2	7.6	0.2	28.2	26.0	30.0	1.8	0	0	0	0
Channel water (*n* = 3)	7.4	7.2	7.4	0.1	25.3	25.1	25.7	0.3	54.4	38.2	75.0	18.8
Treatment plant (*n* = 1)	7.7	7.7	7.7	–	25	25	25	–	4.5	4.5	4.5	–
Water cooler (*n* = 9)	7.6	7.3	7.8	0.2	23.6	20.0	25.8	2.7	3.9	0	22.0	6.9

*SD* standard deviation, *min* minimum, *max* maximum

Turbidity is a standard measurement in water sampling where suspended sediment plays an important role and may be useful for estimating *E. coli* in water particularly for lake water and reservoirs. In Sudan, turbidity of waters can be caused by air molecules where the air is dusty. Turbidity was observed to be 5500 NTU and 45.4 NTU for river and irrigation canal water, respectively (Table 3). These high measurements may indicate the presence of pathogenic microorganisms in these water sources.

Temperature is reported to influence the rate of chemical reactions. The mean values for temperatures ranged from 23.6 to 28.2 °C for all samples collected. The temperature of cooler containers was seen as 23.6 °C. High temperature of 28.2 °C in bottled water can be explained by the fact that the data were taken at midday when temperatures are usually very high in Sudan.

### Water quality of sources

The results showed that 69% of samples collected (84/122) were safe, 14% intermediate (17/122), 6% in high risk (7/122), and 12% unsafe (14/122) water (Table 4). Water sources contained *E. coli* bacteria with variable degrees. River and irrigation canals water were completely unsafe for consumption. The reason might be that these sources are open and exposed to pollutants. Zamxaka et al. (2004) found that more turbid sources are the most microbiologically contaminated. The results showed an association between high turbidity in rivers and irrigation canals and the presence of *E. coli*. Water quality might vary rapidly, due to the rainfall that can greatly increase the levels of microbial contamination in open sources. Hunter (2003) found that waterborne outbreaks often occur following rainfall.

**Table 4:** Water sources with WHO water quality risk categories

Water source	WHO water quality risk categories			
	Safe 0 MPN/100 mL	Intermediate 1–10 MPN/100 mL	High risk 11–100 MPN/100 mL	Unsafe > 100 MPN/100 mL
Yard tap (*n* = 4; 3%)	3 (75%)	–	–	1 (25%)
Communal tap (*n* = 9; 7%)	5 (59%)	1 (11%)	–	3 (23%)
Household tap (*n* = 40; 33%)	26 (65%)	8 (20%)	2 (5%)	4 (10%)
Tank water (*n* = 11; 9%)	7 (64%)	2 (18%)	1 (9%)	1 (9%)
River (*n* = 1; 1%)	–	–	–	1 (100%)
Borehole water (*n* = 39; 32%)	32 (82%)	4 (10%)	2 (5%)	1 (3%)
Channel water (*n* = 3; 3%)	–	–	1 (33%)	2 (67%)
Treatment plant (*n* = 1; 1%)	1 (100%)	–	–	–
Water cooler (*n* = 9; 7%)	5 (56%)	2 (22%)	1 (11%)	1 (11%)
Vendor water (*n* = 5; 4%)	5 (100%)	–	–	–
Total = 122 (100%)	84 (69%)	17 (14%)	7 (6%)	14 (12%)

This result is in line with Valipour (2014) who stated that the increase in irrigation systems has adverse impacts on quality of water resources. Some farmer communities living inside the Gezira irrigation scheme area used canal water for drinking and washing. Boelee et al. (2007) confirmed that in irrigation systems all over the world, water is not only used for the irrigation of agricultural crops, but for a whole range of domestic and other purposes as well. Most of the industrial cities (Wad Medani industrial area) and sugar factories around the study area (Sennar sugar factory) discharge their waste into the river which could have serious adverse health effects on the communities using water for domestic purposes.

A total of 65% of household water taps, 75% of yard taps and 59% of communal taps were safe. Taps normally takes water either from treatment plants through the network distribution of boreholes. The water treatment plant was found to be 100% *E. coli* free, and 82% of the boreholes contained safe water. Boreholes indicated safe groundwater. This result did not agree with results published by Ell-Amin et al. (2010) which indicated that *Wad Medani* groundwater was highly contaminated. This research was conducted in the *Wad Medani* locality, while Amira’s work included the *Managil* locality which is known for polluted water. A study carried out by Engström et al. (2015) in Juba indicated that an important contamination mechanism was fecal pollution of the contributing groundwater, which was probably due to the presence of latrines. Groundwater aquifers in these areas are not rich and communities depend mainly on surface water, which is liable to contamination. Elevated tanks are found either in households or in public schools and are well controlled and routinely checked, and it could therefore account that 64% tanks were containing safe water (Table 4).

### Risk category according to urban, peri-urban and rural areas

In this study, urban population are those living within the *Wad Medani* city, while the peri-urban communities are those living around the city and rural communities are living in villages away from the city where most of them practice agriculture. Table 5 shows the quality of water sources in urban, peri-urban and rural areas of the study area.

**Table 5:** Location with water sources with WHO water quality risk categories

Water source	Urban area				Peri-urban area				Rural area			
	Safe	Intermed	High risk	Unsafe	Safe	Intermed	High risk	Unsafe	Safe	Intermed	High risk	Unsafe
Yard tap (*n* = 4)	3			1								
Communal tap (*n* = 9)	2			2					3	1		1
Household tap (*n* = 40)	15	4		3	3	2			8	2	2	1
Tank water (*n* = 11)	1	1							6	1	1	1
River water (*n* = 1)				1								
Borehole water (*n* = 39)	13	2			12	1			7	1	2	1
Vendor water (*n* = 5)	5											
Channel water (*n* = 3)							1					2
Treatment plant (*n* = 1)	1											
Water cooler (*n* = 9)	5	2		1			1					

Safe = 0 MPN/100 mL, intermediate = 1–10 MPN/100 mL, high risk = 11–100 MPN/100 mL, unsafe ≥ 100 MPN/100 mL

Generally, water sources in urban area are safe particularly treatment plant and bottled water (vendor water) (Table 5). There was, however, tap water which was classified as unsafe for drinking water in the urban area. The river water was totally unfit for drinking water as was one water cooler. Boreholes were found in all areas and generally safe except for 3 boreholes in the rural communities. Water tanks in rural areas were 54.5% safe. Canal water is extended through peri-urban and rural areas. The majority of the rural people are cultivators (Musa et al. 1999) with fields irrigated by water drawn from the canals which is unsafe. The canal water was 33.3% high risk in peri-urban and 66.7% unsafe in rural area. The result shows that the highest level of contamination of water sources (high risk and unsafe) was observed in rural area (9.1%) followed by urban (5.7%), and peri-urban (1.6%).

## Conclusion

Quality of water sources is highly affected by industrial and factory wastes coming from upstream *Wad Medani*. The most contaminated sources are those that are open, turbid and exposed to pollution. Overall the CBT gave fast results and provided a general indication of the water quality in the study area. Frequent and routine qualitative analysis of water sources using the CBT is recommended in Sudan to improve human health and development in areas like *Wad Medani* with little or no resources. Shift to integrated water resources management approach, where water quality is highly considered, will lead to better and sustainable protection of water sources.
